# Explore Awareness of Information Security: Insights from Cognitive Neuromechanism

**DOI:** 10.1155/2015/762403

**Published:** 2015-10-26

**Authors:** Dongmei Han, Yonghui Dai, Tianlin Han, Xingyun Dai

**Affiliations:** ^1^School of Information Management and Engineering, Shanghai University of Finance and Economics, 777 Guoding Road, Shanghai 200433, China; ^2^Shanghai Financial Information Technology Key Research Laboratory, 777 Guoding Road, Shanghai 200433, China; ^3^Shanghai Foreign Language Education Press, Shanghai International Studies University, 550 West Dalian Road, Shanghai 200083, China; ^4^School of Management, Fudan University, 220 Handan Road, Shanghai 200433, China

## Abstract

With the rapid development of the internet and information technology, the increasingly diversified portable mobile terminals, online shopping, and social media have facilitated information exchange, social communication, and financial payment for people more and more than ever before. In the meantime, information security and privacy protection have been meeting with new severe challenges. Although we have taken a variety of information security measures in both management and technology, the actual effectiveness depends firstly on people's awareness of information security and the cognition of potential risks. In order to explore the new technology for the objective assessment of people's awareness and cognition on information security, this paper takes the online financial payment as example and conducts an experimental study based on the analysis of electrophysiological signals. Results indicate that left hemisphere and beta rhythms of electroencephalogram (EEG) signal are sensitive to the cognitive degree of risks in the awareness of information security, which may be probably considered as the sign to assess people's cognition of potential risks in online financial payment.

## 1. Introduction

Today's society is an information society. More and more people use information technologies in daily life and work. They are facilitated by increasingly diversified portable mobile terminals, online shopping, and social media in information exchange, social communication, and e-business. However, when people are enjoying the convenience from information technology, it is also facing the new severe challenges of information security, such as internet intrusion, sensitive information leak, and online payment fraud.

It is well known that information security is a complicated and systematic problem associated with technology, management, economy, and behavioral culture. Up to now, there are a lot of researches on this issue. Cavusoglu et al. studied risks related to information security; they pointed out that risks may have dire consequences, including corporate liability, monetary damage, and loss of credibility [1]. Ensuring information security has become one of the top managerial priorities in many organizations [2–4]. Kuner et al. took the PRISM project as an example which showed that both the offline and online activities had been reported to be related with extensive privacy; they argued that both privacy and security should be protected with individuals' confidence in the rule of law [5]. Numerous studies have shown that the biggest hidden danger of enterprise information security is the internal staff, rather than software vulnerabilities, and employees are often the weakest link in information security [6, 7].

In fact, many information security incidents are not all caused by technology, which happened often due to management oversights or people's weak awareness of information security. For example, behavior of weak password, neglecting the operating system patch, and free use of unsafe mobile devices are related to the lack of recognition of the potential risks on information security. Since the awareness of information security depends on brain cognition of potential risk, it is very important to study brain cognition. A lot of scholars have made great achievements in cognitive research based on cognitive neuromechanism. Qin and Han assessed the neurocognitive processes involved in environmental risk identification by using event-related potential (ERP) and functional magnetic resonance imaging (fMRI); their findings show that an early detection in the ventral anterior cingulate cortex and a late retrieval of emotional experiences in posterior cingulate cortex can help identify dreadful environmental risks [8]. Wang et al. designed and evaluated the vocal emotion of humanoid robots based on brain mechanism; they found that stimulation from audio is related to some brain regional [9]. Dai studied the mechanism of public cognitive emotions when emergencies burst; he pointed out that it needs to consider the public psychology and cognitive ability and that it is easy to accept the way when the city emergency incident bursts out [10]. In addition, some scholars have done the research of brain cognition on investment behavior, framing effect, and microblog information spreading [11–13].

In our study, in order to explore the new technology for the objective assessment of people's awareness and cognition on information security, this paper takes the online financial payment as example and conducts an experimental study based on the analysis of electrophysiological signals.

This paper is organized as follows. In Section 2, the theory and method of cognitive model and EEG are presented. Then, trial is introduced in Section 3. Analysis and results are shown in Section 4. Finally, we provide a summary and discussion about our work in Section 5.

## 2. Theory and Methodology

Awareness is the human mind to reflect the objective material world, and it is the comprehension of feeling, thinking, and other psychological processes. In other words, awareness is a response to a stimulus of human brain. In order to study the information security awareness, cognitive psychology and EEG were used as the research theory and methods.

### 2.1. Cognitive Mechanism of Information Security

#### 2.1.1. Cognitive Psychology

**Figure 1:**
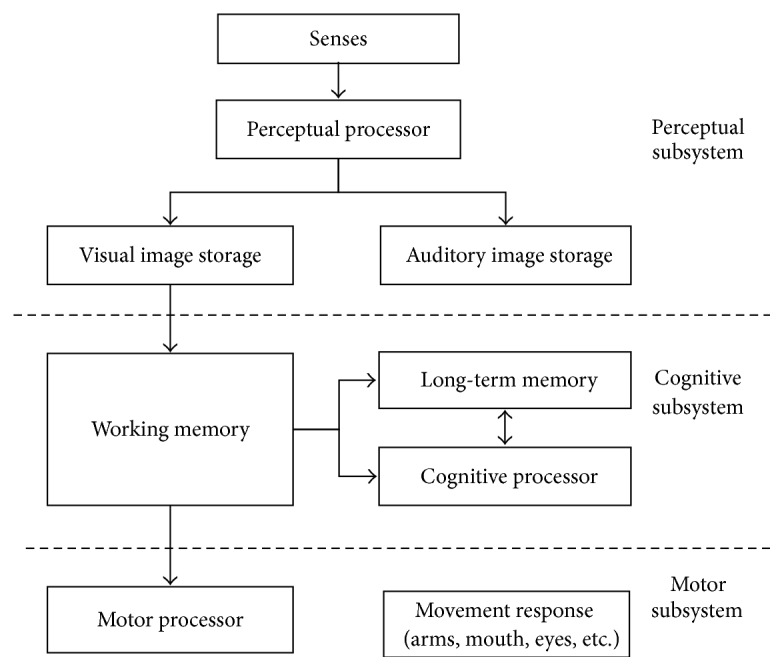
Model Human Processor.

Cognition refers to all processes by which the sensory input is transformed, reduced, elaborated, stored, recovered, and used [14]. Cognitive psychology usually takes human cognitive process as its major subject. It studies the cognitive activities from the viewpoint of information processing, including how humans learn, percept, imagine, memorize, and think of problems. So cognitive psychology is also called information processing psychology. Gagne is a famous scholar in the information processing theory, well-known for his outstanding contribution to information processing model of learning theory. In Gagne's theory, the learning processing was divided into eight stages, and each stage requires different information processing. Firstly, environmental stimuli affected learners; then these stimuli were encoded and were stored as image in the sensor register. These memory images can only store hundredths of a second. Then information entered short-term memory and was encoded again. It can maintain 2.5~3 seconds in here. However, short-term memory is limited to about seven “chunks” of information for most people. Once it exceeds this number, new information will replace the original information. In order to keep the original information, you can repeat it continuously. In this way, information in short-term memory can keep for a long time, but not more than one minute. Finally, the information entered long-term memory and it was encoded again. The majority of people believe that the long-term memory can be stored for a long time. Once you need to use this information, you can retrieve it from long-term memory. In here, information can directly enter response generator, or it can go back to the short-term memory. Meanwhile, expectation and executive control also affected this learning model [15]. After Gagne proposed information processing model, Model Human Processor (MHP) was presented and was used in cognitive modeling. Due to the fact that MHP can calculate the processing time after performing a certain task, it is especially suitable for our study. The processing of MHP is shown in Figure 1 [16]. It can be seen that MHP includes three subsystems, and each subsystem has its own processors and memories.

#### 2.1.2. Cognitive Framework for Information Security Awareness

**Figure 2:**
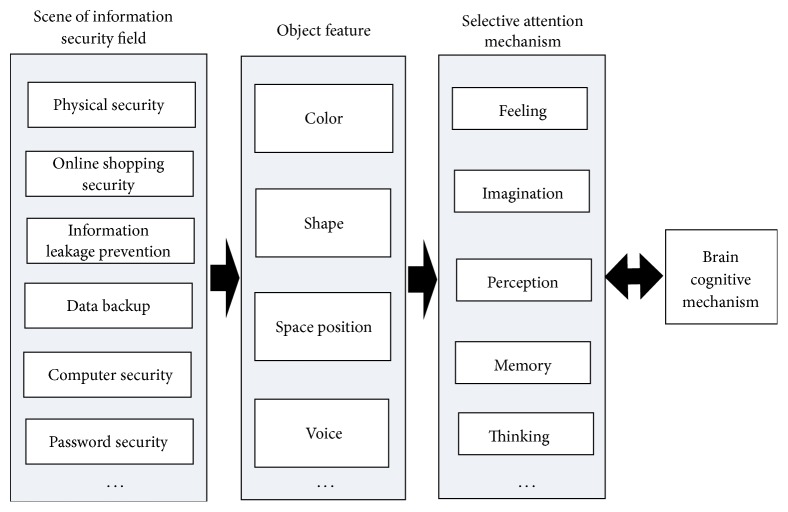
Cognitive framework for information security awareness.

We know information cognition can be viewed as a process of information processing from the previous section. Previous research shows that visual stimuli can produce perceptual awareness [17–19]. Then, visual stimulation of information security was used in our study. And cognitive framework for information security awareness is shown in Figure 2.

From Figure 2, it can be seen that brain cognitive mechanism is closely related to selective attention. For example, when a person feels stimulation from field of information security, such that someone is surfing the internet with the public WiFi or somebody's computer does not install firewall, in the above scene, his brain starts to extract object features of the scene, and the selective attention mechanism begins running, which includes feeling, imagination, perception, and memory. Meanwhile, awareness is also accompanied by brain cognition mechanism which starts running.

### 2.2. EEG Signals Analysis

#### 2.2.1. EEG Waves

The living human brain will continue to discharge, known as electroencephalogram (EEG) [20]. Brain and changes of electricity are the real time performance of brain activity. Generally, the level of volatility reflects brain excitability, and latency reflects the mental activities and processing speed and time evaluation. Human's brain waves frequency range is 0.1~100 Hz, and the frequency and amplitude of four basic brain waves are shown in Table 1 [21].

EEG is closely related to human consciousness, and amplitude of EEG rhythm will increase or decrease when the brain activity increases. Previous research has suggested that *α* rhythms will appear in a relaxed state, *β* rhythms will appear in excited state, *θ* rhythms will appear in drowsy state, and *δ* rhythms usually appear in deep state [21].

#### 2.2.2. EEG Signal Process

EEG signal process mainly includes data cleaning, denoising signal, feature extraction, and classification process. Among them, denoising signal and feature extraction algorithms include power spectrum density estimation, wavelet transform (WT), public space model, multidimensional statistical analysis, and model descriptor. Classification methods include Fisher's linear discriminant, Bayesian method, back-propagation neural network [22], and support vector machine. In our study, WT was used.

WT is a multifunctional multiscale analysis and filter based on combination of time-frequency analysis tool. It has the characteristic of multiresolution and can observe different detail by choosing different basic wavelet, which makes the wavelet transform have the ability to characterize the local features of the signal in the time domain and frequency domain at the same time. Wavelet transform includes Continuous Wavelet Transform (CWT) and Discrete Wavelet Transform (DWT). CWT can be defined as follows:

(1) ψa,bt1a∫−∞+∞ftψ∗t−badt=1aψt−ba,

where *ψ*(*t*) ∈ *L* ^2^(*R*), *a*, *b* ∈ *R*, *a* ≠ 0, then *ψ*(*t*) is called basic wavelet, and *a* means expansion factor and *b* means translation factor.

For the discrete case, DWT can be defined as follows:

(2) $$\begin{array}{c} \psi_{f}\left(\;m,n\;\right)=\overset{+\infty }{\underset{-\infty }{\int }}f\left(\;t\;\right)\psi_{m,n}^{*}\left(\;t\;\right)d\left(t\right), \end{array}$$

where *ψ*(*t*) ∈ *L* ^2^(*R*), *m*, *n* ∈ *Z*.

In order to get high quality EEG signals for analysis, we adopt Discrete Wavelet Transform method and Mallat algorithm to renoise initial EEG signals. Mallat decomposition algorithm is shown as follows:

(3) f0f1+d1=f2+d2+d1=⋯=fN+dN+dN−1+⋯+d2+d1,

where *f*_0_ means initial signal, *f*_*N*_ is the result of the approximation signal after decomposition (low frequency components), and *d*_*N*_ is the result of the error signal after decomposition (high frequency components).

## 3. Experiment

**Figure 3:**
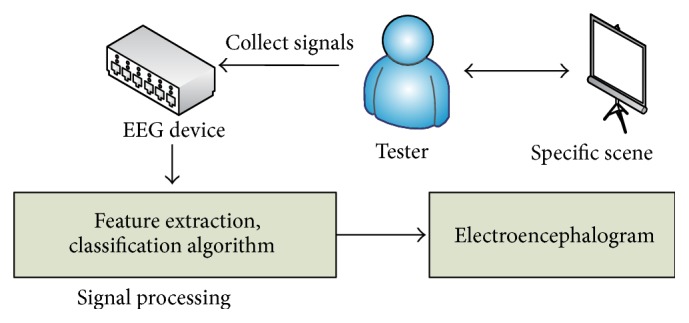
The formation process of EEG.

The formation process of EEG in our trial is shown in Figure 3.

**Figure 4:**
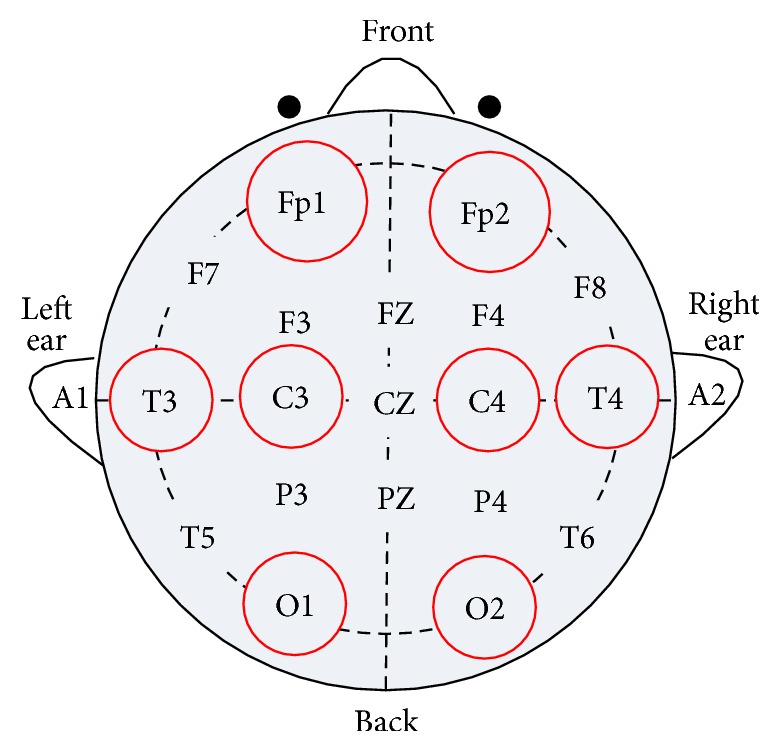
EEG electrodes location of international 10–20 system.

From Figure 3, we can see experimenter watching specific scene and EEG device collecting EEG signals from experimenter. Once collecting signals finishes, the signal process begins to work, and EEG would be shown finally. EEG signal acquisition settings are as follows:

- sampling frequency: 128 Hz;
- amplitude-frequency characteristic: 0.53 Hz–60 Hz;
- electrode placement criteria: electrodes were placed according to the international 10–20 system [23], which is shown in Figure 4;
- electrode channel selection: we choose eight positions of electrode as follows: frontal region (Fp1, Fp2), parietal region (T3, T4, C3, C4), and occipital region (O1, O2) [24];
- using a single-stage lead.

### 3.1. Experimental Overview

Our research involved human subjects, and we recruited 12 healthy adults to participate in our trail; among them, four had received information security awareness training, and eight had not received training. All of their education degrees are bachelor degree or above, with no history of mental illness. They were right-handed with an average age of 27.1 years and they represented 5.69 of the variance. The testing process was told to them before the experiment, and the agreement was signed.

### 3.2. Experimental Design

**Figure 5:**
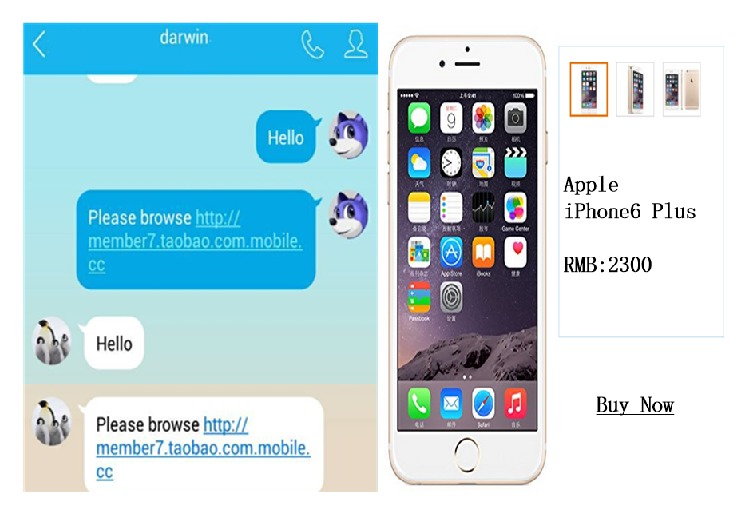
Sample pictures of trail.

In order to research the human awareness of information security, nine experiment scenes were designed in our trial. Testers would make a choice when they take note of information security related pictures or hear fraud words. Tester may encounter fraud information in instant messaging, or access fishing website, or receive fraud text message in his mobile phone, or receive fraud message while using the online payment, and so forth. All of the above scenarios can be used as experimental scene, and sample pictures of trail are shown in Figure 5.

The above website has two suspicions. Left graph uses this link http://www.shbillow.cn/index.mobile.cc.htm, to which the suffix “mobile.cc” was added, and it may be a fishing site. Right graph attracts customers with low price, and the price is too low for the normal price. Tester's information safety awareness may be arousing when he/she notices these scence.

Our experimental procedures are as follows:

- Tester wears electrode cap and puts electrode well. 8 channel recordings are used for electrode cap; 10–20 electrodes are put on standard position according to the International Institute of EEG. Tester seated in the most comfortable, as far as possible, position to ensure the comfort of the viewing test.
- Tester connects to the computer and opens the EEG signal processing software and then checks whether the software works correctly. If there is no problem, then the experiment begins.
- Tester closes his/her eyes, sits and rests, and calms him/herself, when the brain waves are smooth and then begins to record his/her brain waves signal.
- Picture will be shown on the screen. Tester watches picture and listens to the sound with distance of 1 meter, and he or she responds to the prompt. After testing the current scene, another stimulus will appear at random intervals between 1000 ms and 2000 ms. During the interval, the screen background color is black, and the middle of the screen shows the symbol “+” with white color.

### 3.3. Experimental Records

In our experiment, records include tester number, event number, duration, eight-electrode value, and baseline electrode value. Sample of experimental records is shown in Table 2.

## 4. EEG Signal Process and Analysis

### 4.1. EEG Signal Process

**Figure 6:**
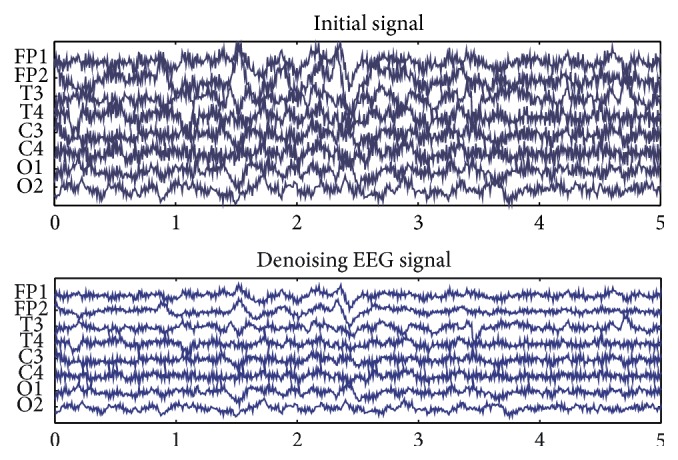
Contrast of initial EEG signal and denoising EEG signal.

Due to the fact that initial EEG signals include a lot of noise, they need to be processed. The process usually includes denoising and characteristics analysis [25]. In order to remove noise signals from the collected EEG signals, we adopted two processes. Firstly, baseline electrode voltage was replaced by the average electrode voltage, and it was recalculated for every electrode voltage. Some noise will be removed after the above steps. Contrast of initial EEG signal and denoising EEG signal is shown in Figure 6.

**Figure 7:**
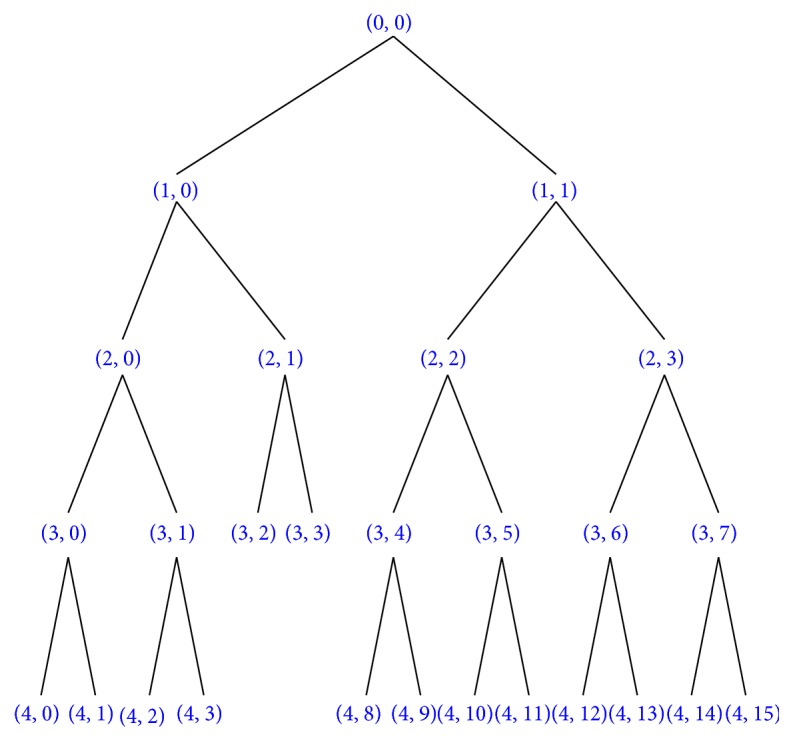
The best wavelet decomposition tree.

Secondly, wavelet transformation method was used for these EEG signals. Because the EEG signal below 30 Hz is worth studying, then we use wavelet filtering to filter above 30 Hz EEG signals. We select the db5 as wavelet packet and decompose EEG signals into four layers. In the process of wavelet decomposition, the best wavelet decomposition tree is shown in Figure 7.

According to sampling frequency which is *f*_*s*_ = 128 Hz, we can calculate frequency width of four layers of each subband as 4 Hz (Δ*f* = (1/2^4^)×(*f*_*s*_/2) = 4), and the four layers include 16-subband wavelet packet *S*(4, *i*), where *i* = 1,2, 3,…, 16. Therefore, four kinds of rhythm waves (*δ*, *θ*, *α*, and *β*) can be extracted by reconstruction. For example, *δ*, *θ*, *α*, and *β* rhythms can be extracted as follows:

(4) $$\begin{array}{c} \delta \left[\;0.5\sim 3.5\;Hz\;\right]\text{: }\left\{\;S\left(\;4,0\;\right)\;\right\},\\ \theta \left[\;4\sim 7\;Hz\;\right]\text{: }\left\{\;S\left(\;4,1\;\right)\;\right\},\\ \alpha \left[\;8\sim 13\;Hz\;\right]\text{: }\left\{\;S\left(\;4,2\;\right)\;\right\},\\ \beta \left[\;14\sim 30\;Hz\;\right]\text{: }\left\{\;S\left(\;4,3\;\right),S\left(\;4,4\;\right),\ldots ,S\left(\;4,7\;\right)\;\right\}. \end{array}$$

### 4.2. Characteristic Analysis

**Figure 8:**
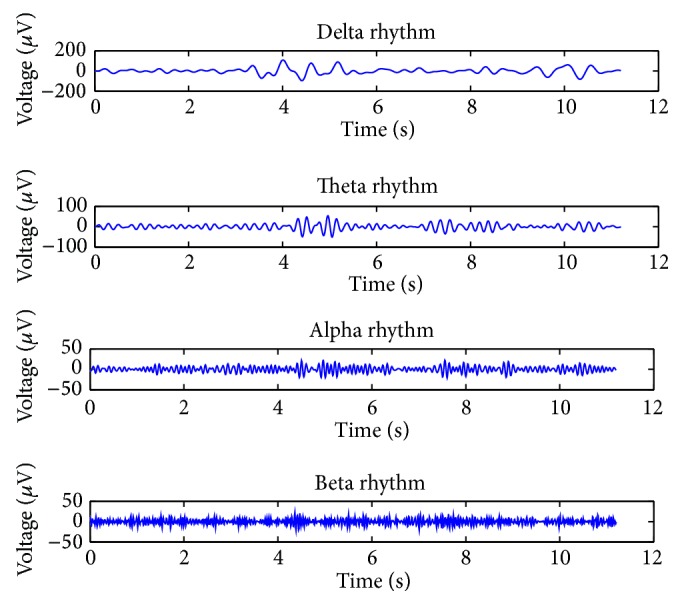
Four types of rhythm signals extracted from wavelet transformation.

In order to analyze the correlation of EEG signal and safety awareness, four types of rhythm signal are extracted from wavelet transformation, which are shown in Figure 8.

In the selection of characteristic parameter, the rhythm energy and energy ratio of four types of rhythm were calculated, and both of them were used for characteristic analysis. Sample of rhythm energy and energy ratio of two test tasks (online payment and online chat) is shown in Table 3.

It can be seen from Table 3 that the alpha rhythm energy and energy ratio are relatively low in two test tasks, which is consistent with previous studies. Previous biomedical research results show that the alpha rhythm became inhibited or disappeared when people are feeling the external stimuli [26]. Our experiment proved that the beta rhythm is consistent with the distribution characteristics of the scalp. It also suggests that beta rhythms are easy to appear when the brain is thinking or exciting. Since information security awareness related to people's focus of attention who remain alert to stimuli for a prolonged period of time, and the beta rhythm is more active, then it can be used to research different brain cognition.

**Figure 9:**
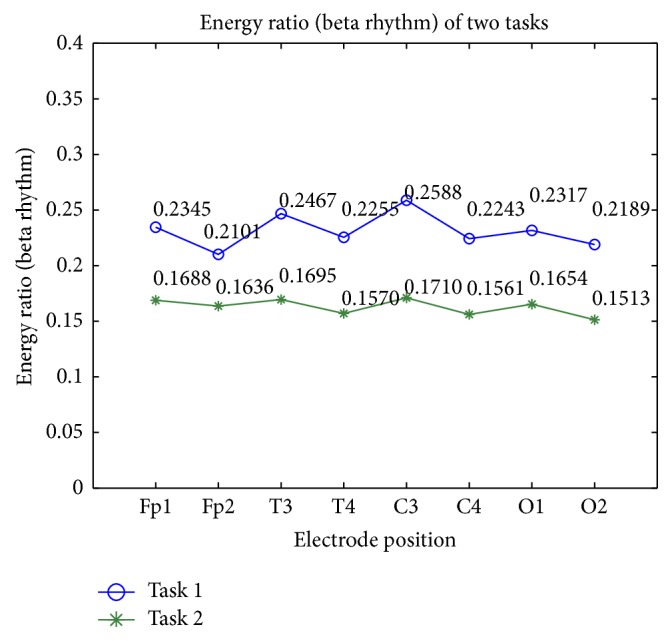
Energy ratio of beta rhythm of two test tasks.

In addition, in order to do a comparative analysis, we choose energy ratio of beta rhythm of two test tasks as comparison; the results are shown in Figure 9. From Figure 9, we can clearly see that energy ratio of beta rhythm of left hemisphere (FP1, T3, C3, O1) is higher than that of the right hemisphere, which shows that the left hemisphere is more involved in reading related tasks.

From Figure 9, we also found that energy ratio of beta rhythm of test task 1 (online payment) is higher than that of test task 2 (online chat). The reasonable explanation is that the tester needs more attention and feels nervous in the online payment than those of the online chat. That is to say, visual stimuli are more likely to arouse the awareness of information security than aural stimuli. Furthermore, the energy ratio of parietal region (T3, C3) is higher than other regions, which showed that the parietal region was involved in awareness of information security related tasks.

In our experimental results, another finding showed that the EEG signals of tester who has been trained on information security were more active than those of untrained tester.

## 5. Discussion

Promotion of people's awareness of information security is the foundation and the precondition of information security of organization. In order to explore the new technology for the objective assessment of people's awareness of information security, this paper conducted cognitive study of information security awareness based on the analysis of EEG signals. We firstly discussed the theory and methodology of EEG signals on cognitive study and then presented a framework for the description of awareness and cognition of information security according to the brain mechanism. On this basis, an experiment was designed to test the reaction of EEG signals to the awareness of hidden problems in information security. This finding showed that the EEG signals could provide a good method for the objective assessment of people's awareness of information security.

In the future studies, we suggest that it can be combined with fMRI (functional magnetic resonance imaging) [27], PET (Positron Emission Tomography), and other measuring equipment to research cognition of individual information security.

## Figures and Tables

**Table 1:** The frequency and amplitude of basic band.

Frequency band	Frequency (Hz)	Amplitude (*μ*V)
*δ*	0.5~3.5	20~200
*θ*	4~7	100~150
*α*	8~13	20~200
*β*	14~30	5~20

**Table 2:** Sample of experimental records.

Tester	Event	Minutes	Seconds	Channel 1	Channel 2	Channel 3	Channel 4	Channel 5	Channel 6	Channel 7	Channel 8	Baseline
Number 01	1	3.511	210.633	33.925	10.482	36.855	−1.352	5.072	7.101	47.675	−135.587	−738.123
Number 01	1	3.511	210.641	25.359	5.410	41.251	11.158	12.172	17.921	74.387	−129.163	−738.123
Number 01	1	3.511	210.648	35.954	31.784	50.719	32.798	27.388	29.417	64.920	−93.322	−738.123
Number 01	1	3.511	210.656	6.875	41.251	32.798	43.618	26.712	36.179	81.826	−66.610	−738.123
Number 01	1	3.511	210.664	53.085	70.330	78.445	78.445	66.948	59.848	120.710	−34.827	−738.123
Number 01	1	3.511	210.672	63.342	68.639	74.049	66.948	65.934	61.877	113.271	−3.381	−738.123
Number 01	1	3.511	210.680	84.869	104.818	130.178	125.782	113.271	113.948	159.594	29.079	−738.123
Number 01	1	3.511	210.688	168.385	151.479	197.802	160.609	167.709	138.292	180.220	51.395	−738.123
Number 01	1	3.512	210.695	194.759	149.789	201.860	160.270	192.392	152.494	207.608	104.480	−738.123
Number 01	1	3.512	210.703	257.875	200.507	253.593	218.090	234.658	196.788	234.996	143.026	−738.123
Number 01	1	3.512	210.711	238.377	187.658	207.270	184.277	180.220	170.752	183.263	123.415	−738.123
⋮	⋮	⋮	⋮	⋮	⋮	⋮	⋮	⋮	⋮	⋮	⋮	⋮
Number 12	9	23.071	1384.227	−28.966	−75.063	−11.158	−52.747	−1.014	−29.417	26.374	−7.439	−738.123

**Table 3:** Rhythm energy and energy ratio.

		Rhythm energy		Energy ratio	
		Task 1	Task 2	Task 1	Task 2
FP1	*δ*	0.3266	0.4593	0.4801	0.5872
	*θ*	0.1386	0.1528	0.2037	0.1923
	*α*	0.0555	0.0396	0.0817	0.0517
	*β*	0.1595	0.1305	0.2345	0.1688

FP2	*δ*	0.3436	0.4734	0.5021	0.5903
	*θ*	0.1525	0.1609	0.2229	0.2007
	*α*	0.0444	0.0364	0.0649	0.0454
	*β*	0.1437	0.1312	0.2101	0.1636

T3	*δ*	0.3304	0.4834	0.4740	0.5988
	*θ*	0.1388	0.1490	0.1991	0.1845
	*α*	0.0559	0.0388	0.0802	0.0472
	*β*	0.1720	0.1361	0.2467	0.1695

T4	*δ*	0.3278	0.4956	0.4927	0.6129
	*θ*	0.1385	0.1523	0.2081	0.1884
	*α*	0.0490	0.0337	0.0737	0.0417
	*β*	0.1501	0.1270	0.2255	0.1570

C3	*δ*	0.3174	0.4906	0.4572	0.6088
	*θ*	0.1402	0.1465	0.2020	0.1818
	*α*	0.0569	0.0390	0.0820	0.0384
	*β*	0.1797	0.1297	0.2588	0.1710

C4	*δ*	0.3370	0.4873	0.5072	0.6102
	*θ*	0.1307	0.1536	0.1968	0.1923
	*α*	0.0476	0.0330	0.0717	0.0414
	*β*	0.1490	0.1247	0.2243	0.1561

O1	*δ*	0.3126	0.4733	0.4833	0.6001
	*θ*	0.1350	0.1472	0.2001	0.1866
	*α*	0.0573	0.0378	0.0849	0.0479
	*β*	0.1698	0.1304	0.2317	0.1654

O2	*δ*	0.3720	0.5257	0.5014	0.6224
	*θ*	0.1482	0.1534	0.1998	0.1816
	*α*	0.0592	0.0377	0.0799	0.0447
	*β*	0.1624	0.1278	0.2189	0.1513

## Dislosure

Yonghui Dai and Xingyun Dai are the joint corresponding authors.
